# Large zooming range adaptive microscope employing tunable objective and eyepiece

**DOI:** 10.1038/s41598-020-71507-8

**Published:** 2020-09-04

**Authors:** Feng-Lin Kuang, Rong-Ying Yuan, Qiong-Hua Wang, Lei Li

**Affiliations:** 1 School of Electronics and Information Engineering, Sichuan University, Chengdu, 610065 China; 2 School of Instrumentation and Optoelectronic Engineering, Beihang University, Beijing, 100191 China

**Keywords:** Microscopy, Adaptive optics, Optofluidics

## Abstract

The conventional microscope has discrete magnification and slow response time in zoom process, which is difficult to capture the dynamic activity of the live specimen. We demonstrate an adaptive microscope employing a tunable objective and a tunable eyepiece with large zooming range. The tunable objective consists of three glass lenses and four electrowetting liquid lenses. The tunable eyepiece consists of an achromatic eyepiece and an electrowetting liquid lens. The focal point between the objective and the eyepiece is designed to be tunable, which are controlled by voltages. Thus, the tuning range is relatively large. We fabricate the adaptive microscope and observe the specimen. In the experiment, the magnification of the microscope changes continuously from ~ 59.1 × to ~ 159.2 × , and the largest numerical aperture is ~ 0.212. The tunable eyepiece can release the back focal length of the tunable objective, which increases the zoom range of the microscope. No mechanical movement is required and the aberrations can be corrected over a wide wavelength range. Thus, the proposed adaptive microscope has a potential application in biological research and clinical medical examination.

## Introduction

Microscopes play an important role in scientific research and production, such as physiological applications^1^, biomedical engineering^2^ and microfabrication^3^. For targets of different sizes, microscopes need different magnifications. Besides, to observe large area of cells and a zoom-in area with high resolution, the higher requirements of real-time observation and continuous zoom change on microscopes emerges^4^. The conventional microscopes can change magnifications by manually converting objectives. But its magnifications are not continuous and the conversion operation introduces sample vibration. One of the solutions for continuous zoom change is to use the mechanical or optical compensation system driven by mechanical movement^5,6^. However, such compensation systems are bulky and sample vibration is still an issue due to the mechanical movement. Besides the slow zoom speed affects the real-time observation. Fortunately, the liquid lenses have changed the traditional lens systems^7^. Due to lightweight, low power consumption and fast response speed, liquid lenses have important applications in imaging^8–10^, display^11^, and communication^12^. Moreover, liquid lenses are wildly used in microscopy as focusing component for axial scanning^13–16^, increasing the depth-of-field^17,18^ and autofocusing^19,20^. However, they cannot achieve continuous zoom change. For example, a five-dimensional microscopy using liquid lens is proposed and able to scan volumes rapidly and reproducibly^21^. This microscopy has fast scan speed however it cannot zoom continuously. A adaptive microscope objective using liquid lens is proposed^22^. The magnification of the adaptive microscope objective tune from ~ 7.8 × to ~ 13.2 × , but its zoom range is very limited due to fixed back focal length (BFL). Therefore, it is still urgent to study microscopes with large zoom range and fast zoom speed.

We demonstrate an adaptive microscope with tunable objective and tunable eyepiece. The tunable objective consists of three glass lenses and four electrowetting liquid lenses. The tunable eyepiece consists of an achromatic eyepiece and an electrowetting liquid lens. The focus of both objective and eyepiece can be adjusted by applying voltage. Different from our previous work^22^, the position of image plane between the tunable objective and eyepiece is flexible, which largely increases the tuning range. We fabricate the adaptive microscope and set up the experiment to observe a resolution chart and a specimen. The magnification of the adaptive microscope changes continuously from ~ 59.1 × to ~ 159.2 × and the largest numerical aperture (NA) is ~ 0.212. The response time is ~ 50 ms. Without introducing mechanical moving parts while zooming, the aberrations can be corrected. The adaptive microscope is suitable for real-time observations that require fast continuous zoom changes.

## Structure and theoretical analysis

**Figure 1:**
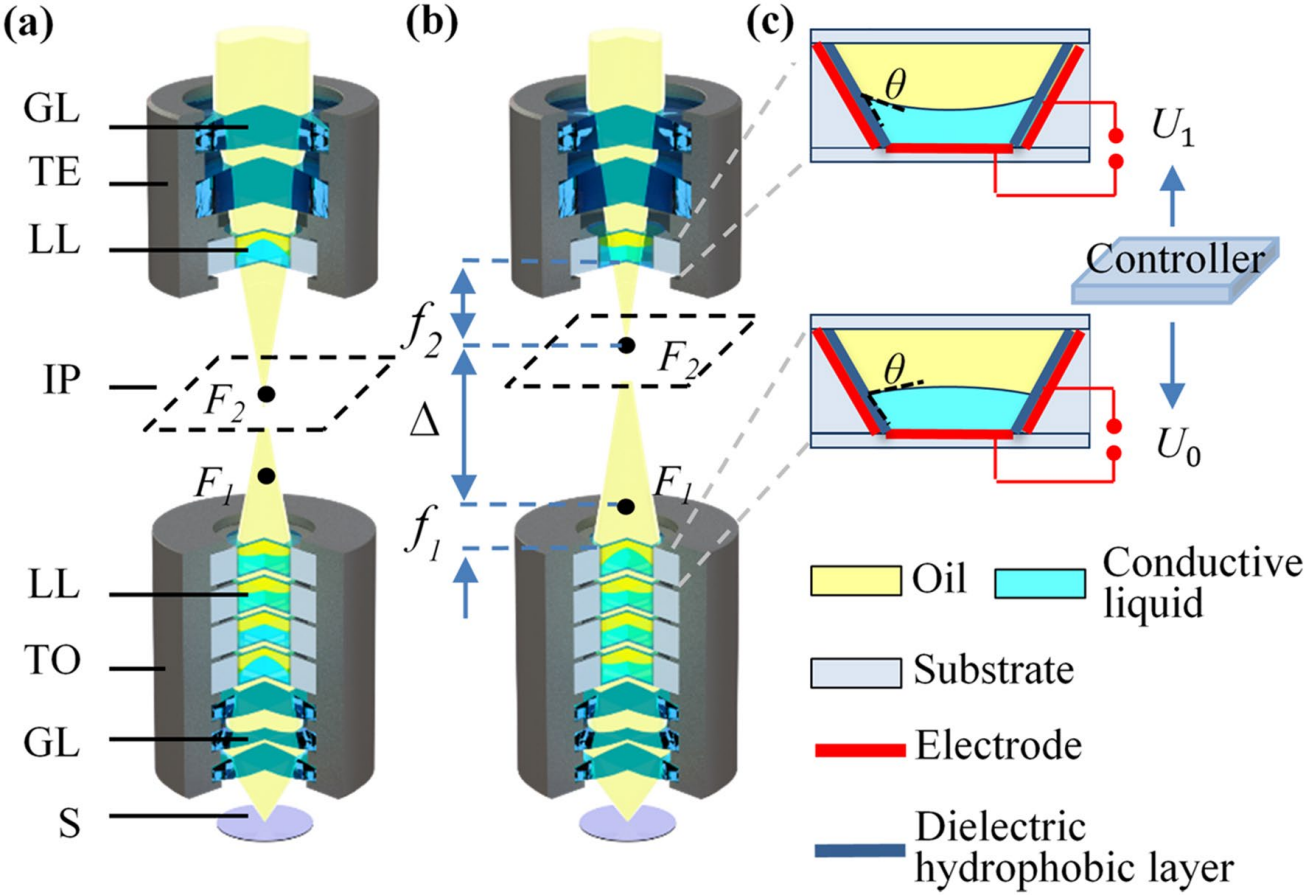
Configuration of the adaptive microscope. **(a)** Initial magnification. *GL* glass lens, *TE* tunable eyepiece, *LL* liquid lens, *IP* image plane, *TO* tunable objective, *S* sample. **(b)** Zoom in. Focal length ${\text{f}}_{1}$ and ${\text{f}}_{2}$ become shorter, and optical spacing ${\Delta}$ becomes longer. **(c)** Liquid lens with different applied voltages, of which ${\text{U}}_{0}$ is lower and ${\text{U}}_{1}$ is higher. θ is the contact angle of the liquid–liquid interface.

A simplified configuration of the proposed adaptive microscope is shown in Fig. 1a. The microscope consists of a tunable objective and a tunable eyepiece, and both are composed of liquid lenses and glass lenses. The dashed box in Fig. 1a, b represent the intermediate image plane of the tunable objective. ${\text{F}}_{1}$ is the focus of the tunable objective and ${\text{F}}_{2}$ is the focus of the tunable eyepiece. ${\text{F}}_{2}$ and the intermediate image plane always overlap.

The design of continuous zoom change is shown in Fig. 1b, where ${\text{f}}_{1}$ is the focal length of the tunable objective, and ${\text{f}}_{2}$ is the focal length of the tunable eyepiece. ${\Delta}$ is the optical spacing between the focus of the tunable objective and the tunable eyepiece. The focuses (*F*_*1*_ and *F*_*2*_) are tuned between the tunable objective and tunable eyepiece. Thus, the focal length ${\text{f}}_{1}$ and ${\text{f}}_{2}$ are changed, which results in the magnification change of the proposed microscope. The magnification of the microscope can be expressed as:

1 $$ \Gamma = - \frac{250\Delta }{{f_{1} f_{2} }}, $$

where 250 represent the distinct vision of 250 mm. When the focal length ${\text{f}}_{1}$ becomes shorter, the magnification of the tunable objective increases, and the imaging plane becomes closer to the tunable eyepiece. To make the focus ${\text{F}}_{2}$ coincide with the intermediate image plane, the focal length ${\text{f}}_{2}$ becomes shorter, which increases its magnification and the optical spacing ${\Delta}$. Thus, the zoom range of the microscope is increased.

Figure 1c shows the structural changes of the electrowetting liquid lens when different voltages are applied. Each liquid lens consists of an oil and a conductive liquid, and can change its optical power by electrowetting effect. θ is the contact angle of the liquid–liquid interface. The relationship between the contact angle θ and the applied voltage ${\text{U}}$ can be described by Young-Lippmann equation^7^:

2 $$ \cos \theta = \cos \theta_{0} + \frac{{\varepsilon U^{2} }}{2d\gamma }, $$

where $\theta_{0}$ is the initial contact angle, γ is the surface tension between the conductive liquid and the dielectric hydrophobic layer, ε is the dielectric constant and ${\text{d}}$ is the thickness of the dielectric hydrophobic layer.

The zoom range of the proposed adaptive microscope increases in two ways. (1) For the tunable objective, the BFL can be adjusted within a certain range instead of fixed, which means the aberrations correction capability of the liquid lenses is released. As a result, the zoom ability of the objective is increased. (2) For the tunable eyepiece, to make the focus ${\text{F}}_{2}$ coincide with the intermediate image plane, the focal length ${\text{f}}_{2}$ changes with the tunable objective. This change increases the magnification of the eyepiece and the optical spacing ${\Delta}$, increasing the zoom range of the microscope. Because the tunable objective plays a decisive role in magnification and image quality, four liquid lenses are used. Two liquid lenses are used to increase the zoom ability, and the other two liquid lenses are used to adjust the position of the image plane and correct aberrations. Using the commercial software Zemax, we constructed a merit function to optimize the radii of four liquid lenses in tunable objective. Then we get the radius solutions that meets the image quality and aberration correction requirements. The radii are converted into applied voltages to achieve continuous zoom change. Since BFL changes while zooming, we adjust the focus of the tunable eyepiece to the intermediate image plane to get the clear image. The tunable eyepiece uses only one liquid lens, because the movement of the BFL is limited, and one liquid lens is enough to adjust its focus to the image plane.

## Fabrication

**Figure 2:**
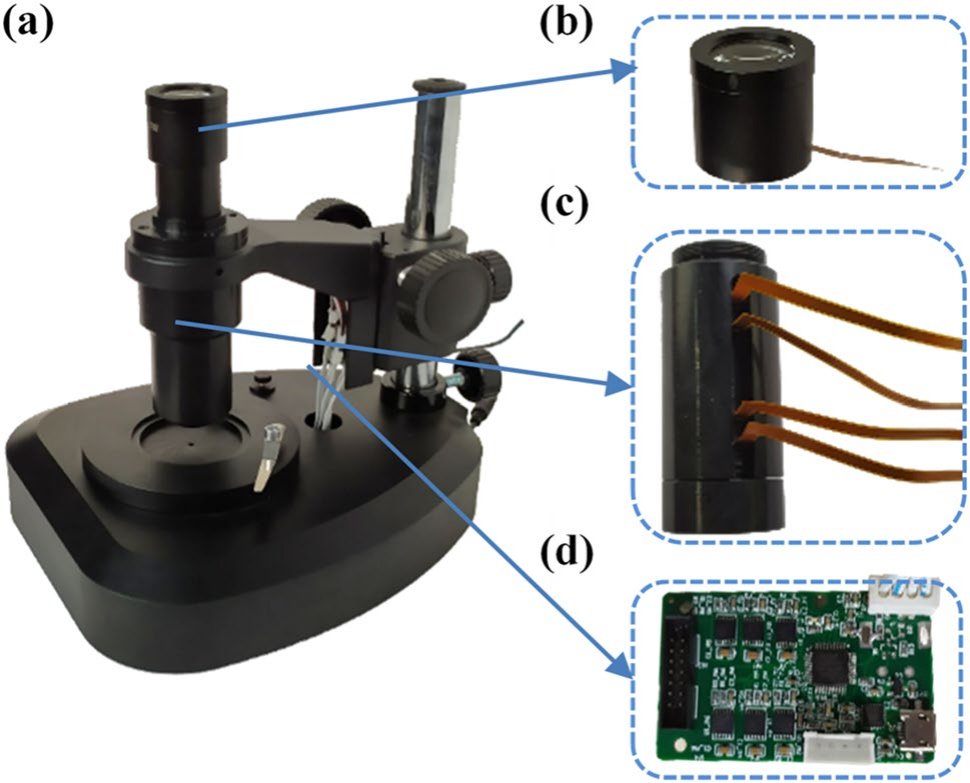
Adaptive microscope and its components. **(a)** The adaptive microscope. **(b)** Tunable eyepiece. **(c)** Tunable objective. **(d)** The controller.

We developed an adaptive microscope shown in Fig. 2a. The microscope consists of a tunable eyepiece, a tunable objective, a controller, a lens cone and a light source. We fabricated a tunable eyepiece as shown in Fig. 2b. The eyepiece consists of a 10 × achromatic eyepiece and an electrowetting liquid lens. Its size is approximately 36 × 36 × 32 (mm).

The fabricated tunable objective is shown in Fig. 2c. Its size is approximately 25 × 25 × 61 (mm), and the focal length ranges from 11.2 to 4.8 mm. For the tunable objective, four electrowetting liquid lenses and three glass lenses are used. The clear aperture of the electrowetting liquid lenses is ~ 4 mm and its main materials are oil and conductive liquid. The diameter of three glass lenses are ~ 6 mm and its material are N-SK16, ZF10 and QK1, respectively. The refractive index and Abbe number of the oil, the conductive liquid and three kinds of glass are shown in Table 1. The controller shown in Fig. 2d is based on ARM 32-bit Cortex M3 CPU (STM32F103ZET6). It provides multiple sets of specific voltages for the adaptive microscope. Each set of voltage includes 5 independent voltage signals. These voltages are applied to the liquid lenses in the tunable objective and the tunable eyepiece, which, according to Eq. (2), results in the change of the focal length of the liquid lenses. Below the target is a LED surface light source (DM-9068^23^) with an adjustable diaphragm.

**Table 1:** Refractive index and Abbe number of the materials used in the tunable objective.

Material	Oil	Conductive liquid	N-SK16	ZF10	QK1
Refractive index	1.500	1.388	1.620	1.689	1.470
Abbe number	34.63	58.13	60.32	31.18	66.87

## Simulation and experiments

**Figure 3:**
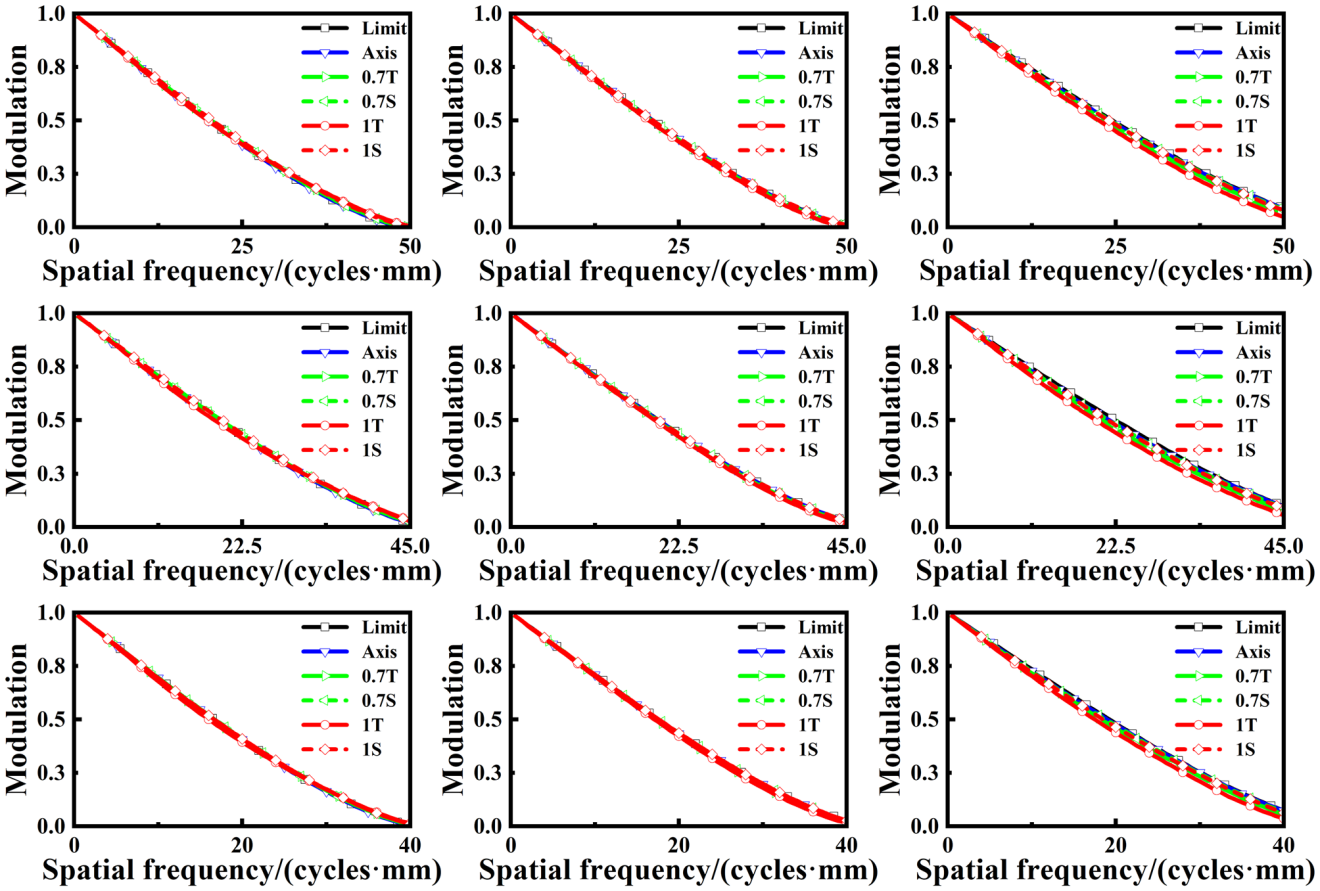
MTF of the proposed microscope. The columns from left to right represent magnifications of 60 × , 110 × , and 160 × , respectively. The lines from top to bottom represent wavelengths of $\text{0.486 }$μm, $\text{0.587 }$ μm and $\text{0.656 }$μm, respectively.

We simulated the proposed adaptive microscope in Zemax within the zoom range of ~ 60 × to ~ 160 × . A paraxial surface was used to simulate the lens of the human eye. The MTF obtained by ray tracing is shown in Fig. 3. The MTF at 60 × , 110 × , and 160 × are given by three wavelengths at $\text{0.486 }$μm, $\text{0.587 }$μm, and $\text{0.656 }$μm, respectively. The black line represents the MTF at diffraction-limited resolution (limit). Axis, T and S represent the zero, tangential and sagittal field of view, respectively. The simulation shows that proposed adaptive microscope can achieve near-diffraction limit resolution at different magnifications and different wavelengths.

We measured the focal length of the electrowetting liquid lens at different applied voltages. The negative focal length varies from ~ − 50 mm to infinity, and the positive focal length varies from ~ 23 mm to infinity. The corresponding negative radius of the liquid–liquid interface varies from ~ − 5.6 mm to infinity and the positive radius varies from ~ 2.7 mm to infinity. Magnification and the NA of the microscope are given in Table 2. The smallest and largest NA are ~ 0.157 and ~ 0.212.

**Table 2:** Detailed optical parameters of the adaptive microscope.

Magnification	60 ×	80 ×	100 ×	120 ×	140 ×	160 ×
NA	0.157	0.169	0.178	0.188	0.196	0.212

**Figure 4:**
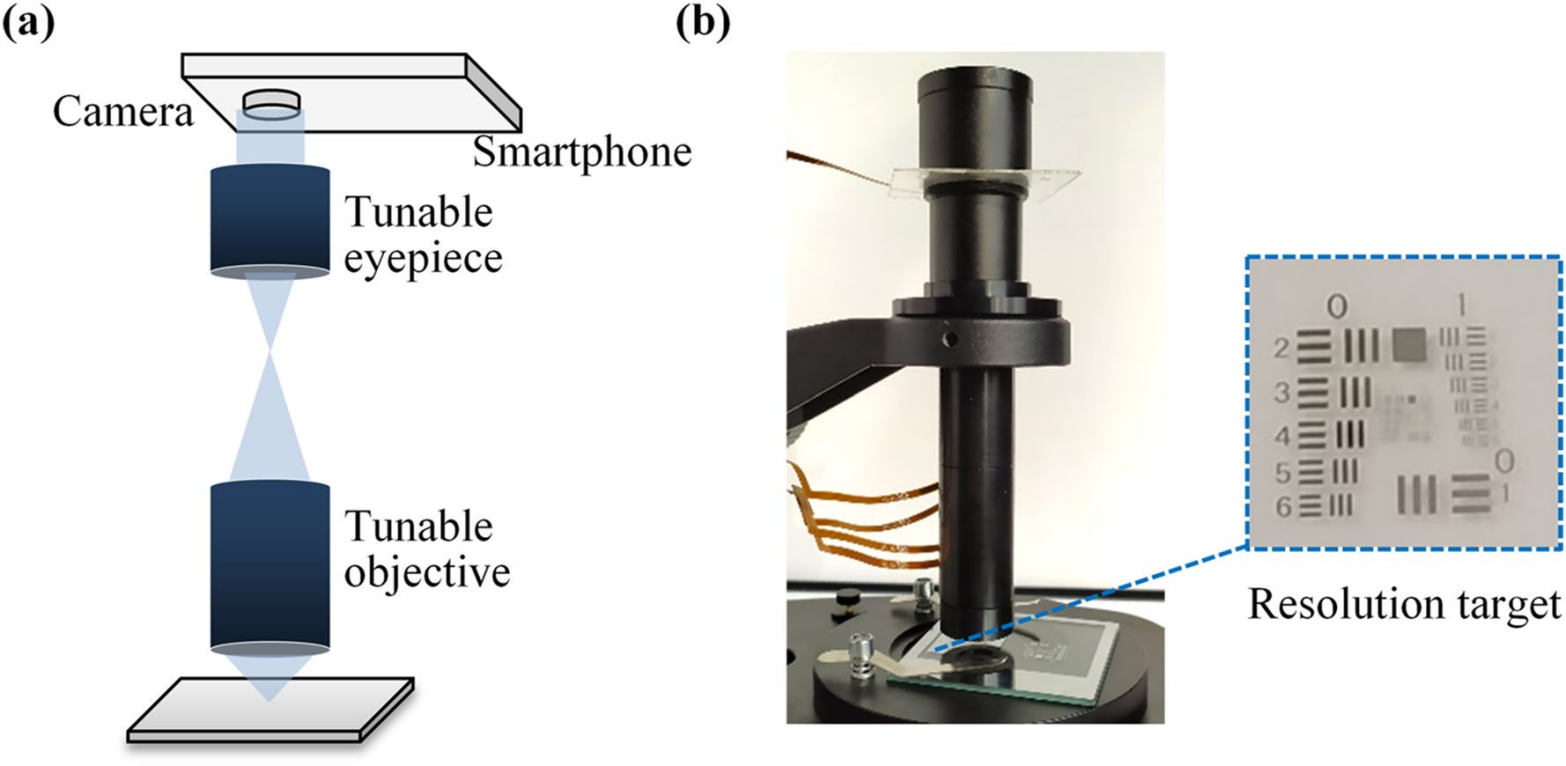
Microscope setup. **(a)** A smartphone camera is used to simulate the human eye. **(b)** Imaging experimental setup. The microscope used directly for visual observation. A USAF 1951 chart is used as resolution target.

The proposed microscope can be used directly for visual observation. To evaluate the imaging quality of the proposed adaptive microscope, we set up an imaging experiment by using a camera of a smartphone to simulate the human eyes, as shown in Fig. 4a. The experiment setup consists of a tunable objective, a tunable eyepiece, a smartphone and a USAF 1951 chart which is used as the resolution target, shown in Fig. 4b. The resolution of the camera (S5KGM1 from Samsung) is 1.6 microns. The focus of the camera is set to a fixed value during the experiment.

For each magnification, firstly, we used the commercial software Zemax to optimize the radii of four electrowetting liquid lenses in the tunable objective. Then the radii were picked up and converted into voltages. When we applied those voltages to the electrowetting liquid lenses, the focal length and BFL of the tunable objective changed. According to the BFL of the tunable objective, we changed the voltage applied to the electrowetting liquid lens in the tunable eyepiece. The focus of the tunable eyepiece was adjusted to the intermediate image plane to obtain clear image. All these voltages were stored in the controller by groups. When we switched between different groups, the microscope can achieve optical zoom.

**Figure 5:**
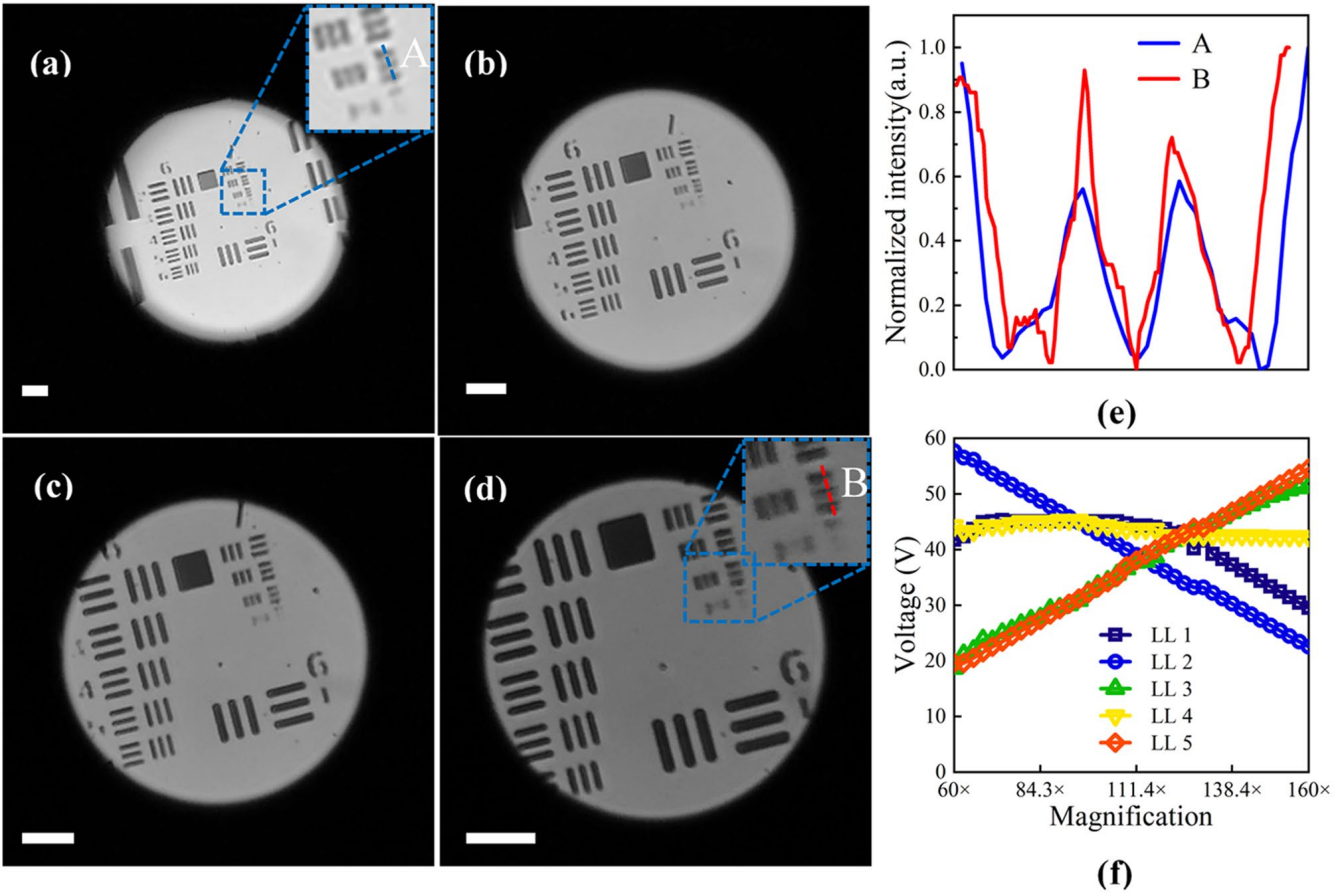
Captured images of the resolution target. **(a)** Zoom 59.1 × . **(b)** Zoom 91.9 × . **(c)** Zoom 119.6 × . **(d)** Zoom 159.2 × . The scale bars in those pictures are 50 μm. **(e)** The blue line **(A)** and red line **(B)** which represent the normalized intensity distribution of the third element of the seventh group at 59.1 × and 159.2 × , respectively. **(f)** Magnification of the microscope versus voltages applied to the liquid lenses. *LL* liquid lens. The liquid lenses in the tunable objective are numbered sequentially from the object side to image side as liquid lenses 1 to 4. Liquid lens 5 represents the liquid lens in the tunable eyepiece.

The captured results are shown in Fig. 5a–d. To obtain the normalized intensity of the resolution target, Fig. 5a–d are converted into grayscale images. The magnification of Fig. 5a is ~ 59.1 × , the entire sixth and seventh group, and part of the fourth and fifth group of resolution target can be seen. When the magnification increases, the resolution target is gradually magnified and the visible parts of the fourth and fifth group are becoming smaller. When the magnification is larger than ~ 119.6 × , as shown in Fig. 5c, only the sixth and seventh group can be seen. The largest magnification is ~ 159.2 × , the captured image is shown in Fig. 5d. In Fig. 5d, the third element of seventh group is clear. From Fig. 5a–d, the visual field becomes larger and the field of view becomes smaller because the focal length of the tunable eyepiece becomes shorter and its magnification becomes larger. The magnification can be linear theoretically. However, the step size of the voltages makes the changes of the magnification in some cases are non-linear. The approximate magnification step size is about 0.1 × . The middle two magnifications show that the microscope can achieve continuous zoom change within a certain range, instead of discrete magnifications such as 10 × , 20 × , 40 × , etc. The color difference between the pictures may be caused by the exposure difference of the images and the NA difference during zooming.

Figure 5e shows the normalized intensity distribution of the third element of the seventh group, where the blue line (A) represents the intensity at Zoom 59.1 × . The modulation of the lines in the center is about 0.19. The red line (B) represents the intensity at Zoom 159.2 × , and the modulation of the lines in the center is about 0.34. The results show that the resolution has been improved by zooming. Figure 5f shows the magnification of the adaptive microscope versus the voltage applied to the liquid lenses in the tunable objective and tunable eyepiece. When the magnification changes, two liquid lenses in the objective work as the zoom part, and the other two liquid lenses are used to adjust the position of the image plane and to correct aberrations. The liquid lens in the eyepiece is mainly responsible for adjusting the focal length of the tunable eyepiece.

**Figure 6:**
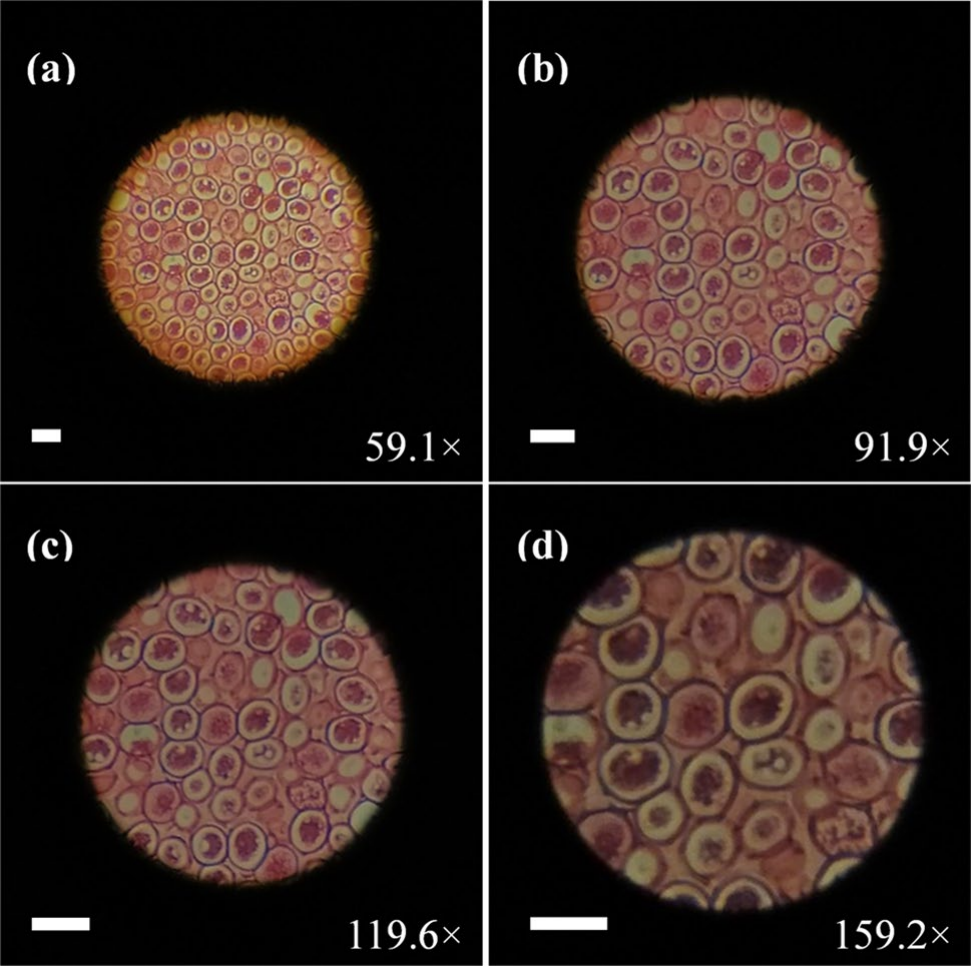
Captured images of the female ascaris sec. When the applied voltage is changed, the cell is continuously enlarged (see Visualization 1). **(a)** Zoom 59.1 × . **(b)** Zoom 91.9 × . **(c)** Zoom 119.6 × . **(d)** Zoom 159.2 × . The scale bars in those pictures are 50 μm.

To verify biological applications of the proposed adaptive microscope, we used a section specimen of female ascaris as the target. The result is shown in Fig. 6. Figure 6 remains unchanged, which makes its color different from that in Fig. 5a–d. From Fig. 6a–d, as we continuously zoom in, the cells of the female ascaris are gradually enlarged and the image remains sharp at each magnification. A video of the different magnification (see Visualization 1) shows that the zooming process is continuous and fast. The response time is measured to be ~ 50 ms. As the focal length of the eyepiece becomes shorter, the visual field becomes larger and the field of view becomes smaller, as we observed in Fig. 5. The lower brightness of the picture in Fig. 6d is due to the lack of light intensity. As the magnification increases, the light intensity needs to be increased to maintain the same brightness of the image, which is similar to the traditional microscope.

We note that there are small vibrations in Visualization 1. There are two reasons for that: (1) Since the optical power of the liquid lenses are controlled by voltages, the vibrations of the voltages are the main reason for the small vibrations. (2) The vibrations of the external environment cause the stage to move up and down, which will also cause the image vibrations.

Since the aperture of the liquid lens in the eyepiece is ~ 4 mm, the field of view of the microscope relatively small. We believe that using a liquid lens with a larger diameter can get a larger filed. However, the microscope will be complicate to operate and zoom speed will be slower, which will affect the real-time observation. The zoom range of our proposed microscope is ~ 59.1 × to ~ 159.2 × . Increasing the optical power range of single electrowetting liquid lens, or using more liquid lenses will increase the zoom range of the microscope. However, more liquid lenses will make the microscope more complicated and will increase the costs. In this microscope, working distance is fixed which means no moving parts are required. However, achieving a larger zoom range at a fixed working distance will be a challenge, because the requirement of optical power range is relatively large. With the development of the liquid lenses, more and more research will be put into the microscopes to achieve larger zoom range. Changing working distance without moving part may address these issues in the future.

## Conclusion

In this paper, we present an adaptive microscope consisting of a tunable objective and a tunable eyepiece. The tunable objective consists of three glass lenses and four electrowetting lenses. The tunable eyepiece consists of an achromatic eyepiece and an electrowetting lens. The focus of the objective and the eyepiece are tunable by changing the applied voltages. Because the focus of the eyepiece always coincides with the intermediate image plane during zooming, the BFL of the tunable objective is released, which increases the zoom range of the microscope. Without introducing mechanical moving parts, the magnification of the microscope changes from ~ 59.1 × to ~ 159.2 × and its response time is ~ 50 ms. Observation of resolution targets and specimens shows that our microscope has the ability of real-time observation with continuous zoom change. In specific application, such as tumor metastasis. It is necessary to observe a relatively large field of view, while being able to magnify the area of interest and to observe the morphology of the cell. Our adaptive microscope is suitable for such applications. Besides, the proposed adaptive microscope has potential applications in physiological research and manufacturing.

## Supplementary information

Supplementary Legend.

Supplementary Video 1.
