# Supplementary material for: Large zooming range adaptive microscope employing tunable objective and eyepiece
Source: Sci Rep. 2020 Sep 4;10:14644. doi: 10.1038/s41598-020-71507-8 (PMC7474087; doi:10.1038/s41598-020-71507-8)
Supplement: Supplementary file 1 — Supplementary Legend. [file 41598_2020_71507_MOESM1_ESM.doc]

**Large zooming range adaptive microscope employing tunable objective and eyepiece**

Feng-Lin Kuang,^1)^ Rong-Ying Yuan,^2)^ Qiong-Hua Wang,^2, *)^ and Lei Li ^1, *)^

**Supplementary Video.**

**Visualization 1.** A video of the different magnification of the adaptive microscope.

**Supplementary Video Legend**

**Visualization 1.** When the applied voltage is changed, the cell is continuously enlarged, which shows that the zooming process is continuous and fast.

^1^ School of Electronics and Information Engineering, Sichuan University, Chengdu 610065, China

^2^ School of Instrumentation and Optoelectronic Engineering, Beihang University, Beijing 100191, China

^*^ Correspondence: Dr. Lei Li, Email: [leili@scu.edu.cn](mailto:leili@scu.edu.cn), Pro. Qiong-Hua Wang, Email: [qionghua@buaa.edu.cn](mailto:qionghua@buaa.edu.cn)
